# Defining a competency framework for health and social professionals to promote healthy aging throughout the lifespan: an international Delphi study

**DOI:** 10.1007/s10459-024-10316-4

**Published:** 2024-03-05

**Authors:** Míriam Rodríguez-Monforte, Carles Fernández-Jané, Marietta Bracha, Adrianna Bartoszewska, Mariusz Kozakiewicz, Mariel Leclerc, Endrit Nimani, Pauliina Soanvaara, Sari Jarvinen, Meike Van Sherpenseel, Miriam van der Velde, António Alves-Lopes, Marietta Handgraaf, Christian Grüneberg, Elena Carrillo-Alvarez

**Affiliations:** 1 Global Research on Wellbeing (GRoW), Blanquerna School of Health Sciences - Ramon Llull University, Barcelona, Spain; 2 Departament deSalut, Universitat Pompeu Fabra, Tecnocampus, Mataró-Maresme, Barcelona, Spain; 3 Department of Geriatrics, Nicolaus Copernicus University, Torun, Poland; 4 Heimerer College, Prishtina, Kosovo; 5 JAMK University of Applied Sciences, Jyvaskyla, Finland; 6 HU University of Applied Sciences Utrecht, Utrecht, The Netherlands; 7 Research Group Innovation of Human Movement Care, Research Center for Healthy and Sustainable Living, HU University of Applied Sciences, Utrecht, The Netherlands; 8 Alcoitão School of Health Sciences, Alcabideche, Portugal; 9 HS Bochum University of Applied Sciences, Bochum, Germany

**Keywords:** Healthy aging, Health professionals, Social professionals, Competencies, Delphi study

## Abstract

The promotion of healthy aging has become a priority in most parts of the world and should be promoted at all ages. However, the baseline training of health and social professionals is currently not adequately tailored to these challenges. This paper reports the results of a Delphi study conducted to reach expert agreement about health and social professionals’ competencies to promote healthy aging throughout the lifespan within the SIENHA project. Materials and methods: This study was developed following the CREDES standards. The initial version of the competence framework was based on the results of a scoping review and following the CanMEDS model. The expert panel consisted of a purposive sample of twenty-two experts in healthy aging with diverse academic and clinical backgrounds, fields and years of expertise from seven European countries. Agreement was reached after three rounds. The final framework consisted of a set of 18 key competencies and 80 enabling competencies distributed across six domains. The SIENHA competence framework for healthy aging may help students and educators enrich their learning and the academic content of their subjects and/or programs and incentivize innovation.

**Supplementary Information:**

The online version contains supplementary material available at 10.1007/s10459-024-10316-4.

## Introduction

The promotion of healthy aging has become a priority in most parts of the world. Once believed to solely concern older adults, there is growing consensus that healthy aging should be promoted at all ages (Chen, 2016; Michel et al., 2016).

On 15 November 2022, the world population was estimated to have reached 8 billion. However, the European population is expected to shrink by 30.8 million (− 6.9%) from 2019 to 2100, with an uneven reduction across age groups. Furthermore, the share of people aged 65 and over is likely to increase by 11%, reaching a total of 130.2 million by 2100 (Eurostat, 2023).

Such projections more than 70 years ahead provide a unique opportunity to plan for future realities. The United Nations (2022) urged regions with aging populations to take steps to prepare their countries for the changing needs of the aging population. Preparing for aged societies involves arranging for the needs of the larger over-65 population and specifically deploying the means so that people who will be aged 65 in ten, twenty, fifty or seventy years reach old age in the best possible conditions.

Aging starts from conception. The life course perspective examines how intrinsic and extrinsic exposure from the intrauterine medium and throughout a person’s lifetime result in variations in individuals’ rate and quality of aging (Hanson et al., 2016). This approach focuses on various factors, life opportunities and choices that might significantly influence health status, lifestyle, and health and social inequalities in older age and challenges the focus of most research on the topic, which exclusively investigates the effect of influences in the later stages of life (Grundy et al., 2013; Kuh et al., 2013).

Lifestyle factors such as dietary habits, physical activity, smoking and alcohol consumption, psychosocial aspects such as social participation, social support and social networks, adjustment to retirement, and upstream determinants such as social status, education and income have been identified as playing important roles in achieving healthy aging in European populations and should be addressed by policy-makers (Sowa et al., 2016). Differences exist within and between countries and must be taken into account. For example, elderly people living in Western Europe report greater well-being in old age than those in southern and central-eastern European countries, and differences between the sexes are particularly wide in the southern region, with women reporting lower levels of healthy aging.

Sadana et al. (2016) investigated the origins, pathways and opportunities to intervene to reduce inequities in healthy aging. They identified specific areas of intervention for children and adolescents, young adults, adults and older adults (e.g., family policies, early child development activities, labor market and employment conditions, actions to reduce the exposure to risk factors for NCDs and CDs, universal insurance schemes, adequate infrastructures) and cross-sectional actions along the life-course directly related to health and health-care systems. Their results emphasize that the social and health workforce must be better equipped to promote healthy aging throughout the lifespan to satisfy the diverse needs of new older adults from the perspective of health and welfare advocacy as well as organization/management and to avoid reinforcing inequities.

The SIENHA project (Strategic, Innovative, Educational Network for Healthy Aging 2020-1-ES01-KA203-083121) is an EU-funded initiative that aims to contribute to this challenge by transforming understanding and supporting the continuous development of the competencies of students, teachers and professionals to foster healthy aging across Europe. To this end, the project is developing a framework of competencies in healthy aging, a curriculum for healthy aging, and an innovation and research toolkit for healthy aging, all included in a handbook on healthy aging. This paper reports the results of a Delphi study conducted with the aim of reaching expert agreement about health and social professionals’ competencies to promote healthy aging throughout the lifespan.

## Materials and methods

### Study design

The Delphi technique was chosen to identify the relevance and reach consensus among health and social professionals regarding the competencies for healthy aging based on the competency framework constructed by the SIENHA Consortium (Brown, 1968; Jünger et al., 2017). To allow critical appraisal of the methodology and resulting guidance, our study followed CREDES, a reporting standard for Conducting and Reporting of Delphi Studies (Jünger et al., 2017).

### Competency framework construction

The initial step of the SIENHA Project involved a scoping review to identify and reflect on the competencies for health and social professionals related to healthy aging complemented by stakeholder meetings with experts within the field of healthy aging (Carrillo-Alvarez, 2023). This initial step was followed by discussion sessions within the SIENHA Consortium involving critical dialog and debate led by two experts in pedagogy with the aim of operationalizing the competencies identified using the CanMEDS competency framework (Royal College of Physicians & Surgeons of Canada, 2015). CanMEDS is an internationally recognized framework used as a basis for educational and practice standards in many countries. Originally, it was used in medical education; however, it has also been used to define competency within various health professions (Dijkman et al., 2016).

Health and social professionals have their own body of knowledge, skills and attitudes. When focusing on healthy aging, the emphasis, in addition to the primary focus of one’s own profession, is the prevention of health problems associated with aging throughout the lifespan, in the broadest sense of the term. Thus, the focus on healthy aging is reflected on an individual level and at the level of communities and/or populations. Based on this idea, the key competencies and enabling competencies are described and integrated into the different roles of the CanMEDS framework, (1) health and welfare advocate; (2) communicator; (3) collaborator; (4) leader; (5) scholar; and (6) professional, with a focus on the prevention of health problems related to healthy aging. From this perspective of healthy aging, the individual level and the level of communities and populations fit within the role of the health and welfare advocate. Therefore, competencies for the role of an expert were not formulated.

### Design and procedure of the Delphi study

A protocol was designed and approved by the SIENHA Consortium to guide the steps of the Delphi study. Additionally, a steering group was formed that met at key stages to oversee participant recruitment, data analysis, study development and the dissemination of the findings. The steering group consisted of five members, three study investigators and two external members.

#### Delphi study participants

The expert panel consisted of a purposive sample of experts in healthy aging with academic and clinical backgrounds, comprising academic experts with a minimum of two peer-reviewed publications on healthy aging in the past five years and active professionals with a minimum of five years of experience in the same field of practice (clinical or social) and a minimum of five years of experience in research. To establish a representative sample, we followed the criteria established by Jorm et al. (2015), who describe stability in findings with panels of approximately 20 or more members. Considering a 30% rejection rate, 27 experts were invited to participate in the study and were contacted by the different project partners. Twenty-two experts agreed to participate. The participants were unknown to each other but not to the researchers. The panel members were assured that all information would be confidential and would comply with the research data security policies.

The ethics committee of Ramon Llull University approved the study (CER URL_2022_2023_006). All participants’ identities were kept anonymous by using codes. The participants received information about the study with the invitation to participate, and prior informed consent was obtained for participation in this study.

#### Delphi study procedure

**Fig. 1:**
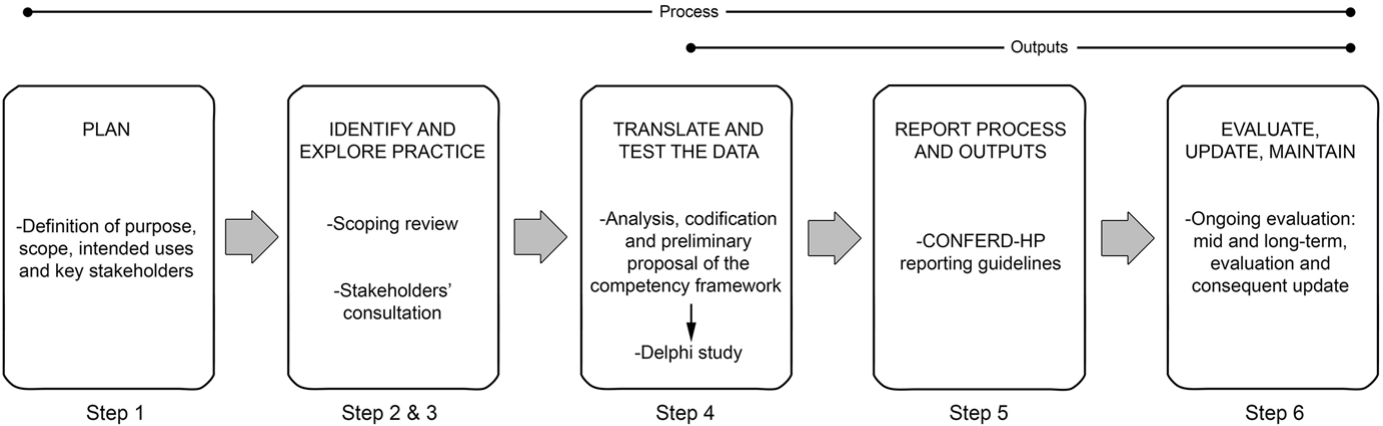
Framework process

The key and enabling competencies were included in a survey form. Using the Welphi Platform (Welphi, 2023), a Delphi process was designed to run until consensus was met or for a maximum of three rounds because response exhaustion usually occurs within several rounds (Fig. 1).

The questionnaire for each round was piloted with the steering group to test readability, relevance, and appropriateness. Feedback and changes suggested by the steering group were agreed upon between the study co-authors before the implementation of the following round.

In the first round, competencies were ranked from high to low relevance using (first round) a five-point Likert scale (1 = not at all relevant (must not include), 5 = very relevant (must include)) in response to the question, “Is this competency relevant to health/social professionals in the provision of healthy aging throughout the lifespan?” Suggestions for additional recommendations and the reasons for them were obtained by including a final section for qualitative input.

**Fig. 2:**
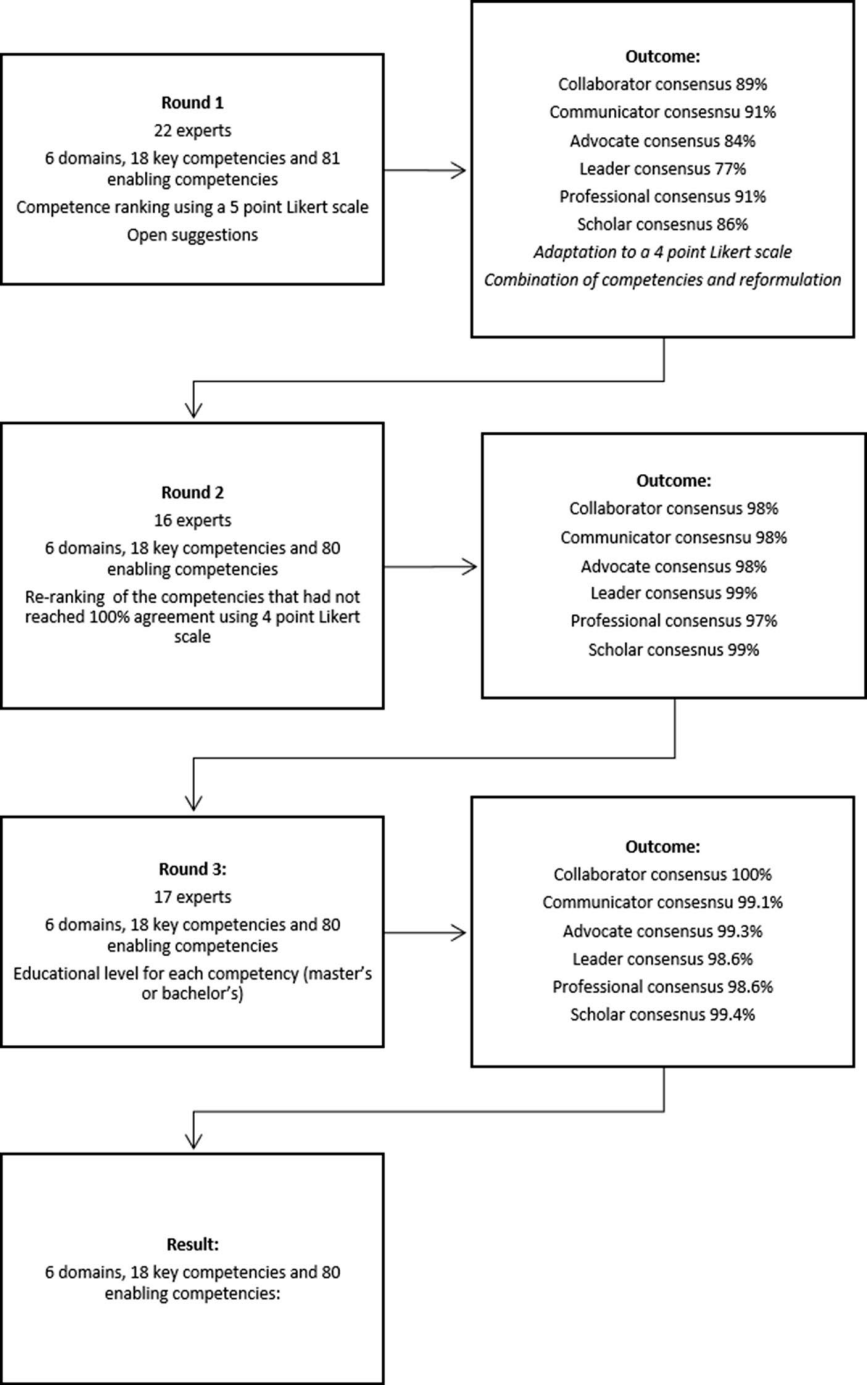
Diagram of the Delphi rounds

After the first round, based on the results and the steering group consensus, the Likert scale was adapted to a four-point scale (1: not at all relevant; 2: not relevant; 3: relevant; 4: very relevant) to avoid middle-range ratings (3 in the 5-point Likert scale), which were very common in the first round and included no additional feedback on the reason for the selection (Fig.2).

The competencies suggested by the participants were analyzed inductively based on the text material. Competencies with the same content were combined, and inadequately described competencies were reworded (Supplemental Online Material).

#### Data collection and consensus definition

Round 1 of the Delphi survey included all competencies specified in the constructed framework and was designed to take less than 60 min to complete. Subsequent rounds took less time to complete as the number of competencies for consideration decreased.

Following the initial invitation, panel members were given 10 days to provide their consent to participate and 15 days to respond between each round. Up to four reminders were sent in each round.

We used 75% agreement on the rating of relevance of the recommendation to determine consensus (Diamond et al., 2014; Gibson et al., 2020).

The ranked competencies were divided into two relevance groups: Group 1, must include (at least 75% of respondents scoring 3 or 4 on the Likert scale) and Group 2, must not include, not relevant (at least 75% scoring 1 or 2).

Competencies rated as “must not include” by 75% or more of the respondents were eliminated from the list. The panel’s aggregate responses for each competency from rounds 1 and 2 were noted beside each competency as a percentage of “must not include” and “must include”. The changes based on the qualitative feedback of the expert panel were pointed out to the participants in rounds 2 and 3 by changing the font color of the modifications (Penciner et al., 2011).

The participants’ demographics (gender, age, country of citizenship, level of education, field of studies, and work) were collected.

The responses from each round were aggregated and fed back to the panel members anonymously in the following round.

### Data analysis

Descriptive statistics were obtained on the demographics of the experts, and each participant was linked to a master code. The statistics also included the mean, median, and standard deviation. For the analysis, we used Welphi and Excel^®^.

The construction of the initial SIENHA Competency Framework based on the CanMEDS Framework as well as the analysis and agreement achieved by the five members of the steering group before each Delphi round were developed through critical dialog and debate within the different teams (McCormack & McCance, 2006).

## Results

### Demographic data of participants

A mean of 18 experts participated in our study (first round: 22; second round: 16; third round: 17). The majority of participants in all rounds were female (first round: 68.18%; second round: 75%; third round: 64.7%). The participants’ mean age was 46.4 years ± 10.8 SD. Their fields of studies comprised nursing, physiotherapy, nutrition and dietetics, medicine, psychology, geriatrics, and social and health care, and the participants’ median experience in the field was 15.5 years (± 4.55 SD).

The participants’ level of education included master’s degrees (30.14%) and doctoral degrees (66.7%), and one participant was a professional fellow.

The demographic characteristics of the participants can be found in Table 1.

**Table 1:** Sociodemographic characteristics of study participants

	R1		R2		R3	
	n	%	n	%	n	%
Participants in each Delphi round (22 participants agreed to participate)	21	95.45	16	72.72	17	77.27
Gender	n	%	n	%	n	%
Female	15	71.42	12	75	11	64.7
Age	Mean	SD	Mean	SD	Mean	SD
	48.1	10.73	45.6	12.7	45.7	9
Country of citizenship	n	%	n	%	n	%
Poland	4	19	4	25	4	23.5
Finland	3	14.2	2	12.5	3	17.6
Germany	0	–	2	12.5	3	17.6
Kosovo	1	4.76	1	6.25	1	5.8
Portugal	0	–	1	6.25	0	–
The Netherlands	4	19	2	12.5	2	11.7
Spain	2	9.5	2	12.5	2	11.7
France	1	4.76	0	–	0	–
Norway	1	4.76	1	6.25	1	5.8
UK	1	4.76	1	6.25	1	5.8
Not reported	4		–		–	
Degree of education	n	%	n	%	n	%
Doctoral studies	NA	NA	11	68.7	11	64.7
Master	NA	NA	4	25	6	35.29
Fellow professional	NA	NA	1	6.25	0	0
Field of studies and work	n	%	n	%	n	%
Nursing	2	9.5	2	12.5	2	11.76
Physiotherapy	3	14.2	4	25	4	23.52
Nutrition and dietetics	2	9.5	1	6.25	2	11.76
Medicine	3	14.2	2	12.5	2	11.76
Psychology	3	14.2	3	18.75	2	11.76
Social work	1	4.76	0	0	1	5.88
Geriatrics	3	14.2	4	25	2	11.76
Other (education, research, sports)	4	19	0	0	2	11.76

*R* Delphi round, *SD* Standard deviation, *NA* Non available

### SIENHA competency framework

Initial competency based on the scoping review conducted by the SIENHA Consortium and the discussion sessions was presented in a framework that included six domains (collaborator, communicator, health and welfare advocate, leader, professional, and scholar) with 18 key competencies and 81 enabling competencies. The final model included the same six domains with 18 key competencies and 80 enabling competencies. In the different rounds, 16 competencies were reformulated and one enabling competency was added (health and welfare advocate; enabling competency 1.8) (Table 2 and Supplemental Online Material).

**Table 2:** Overview of results from the delphi rounds and overall consensus for each round across domains (including key and enabling competencies) and across key competencies of each competency framework domain

	Round 1			Round 2			Round 3		
Domain of the SIENHA Competency Framework on healthy ageing	KC	EC	Consensus achieved *(relevant or very relevant)* %	KC	EC	Consensus achieved *(relevant or very relevant)* %	KC	EC	Consensus achieved *(relevant or very relevant)* %
**COLLABORATOR** As collaborators, health and social care professionals work together with others to promote and support healthy ageing throughout the lifespan among individuals, groups of individuals and/or communities. The forms of collaboration can involve the individuals’ families*, health and social care professionals, community partners and other stakeholders	**2**	**11**	**89**	**2**	**11**	**98**	**2**	**11**	**100**
*KC 1: To be able to work effectively with other professionals within and outside the health and social care profession to promote and support healthy ageing throughout the lifespan*	*Mean* ± *SD*	*3.79* ± *0.41*	*90.47*	*Mean* ± *SD*	*3.87* ± *0.33*	*100*	*Mean* ± *SD*	*3.87* ± *0.33*	*100*
KC 2: To be able to work effectively with individuals and families* to promote and support healthy ageing throughout the lifespan		*3.67* ± *0.74*	*80.95*		*3.75* ± *0.43*	*100*		*3.75* ± *0.43*	*100*
**COMMUNICATOR** **As Communicators, health and social care professionals form positive relationships with the individuals and their families, facilitating the gathering and sharing of essential information related to healthy ageing. Using person-centered communication, they support and advise individuals in shared decision-making and lead effective interactions that promote health and well-being**	**3**	**17**	**91**	**3**	**17**	**98**	**3**	**17**	**99.11**
*KC 1: To be able to communicate effectively with individuals, families and stakeholders to establish strong positive relationships with them*	*Mean* ± *SD*	*3.63* ± *0.48*	*90*	*Mean* ± *SD*	*3.56* ± *0.50*	*100*	*Mean* ± *SD*	*3.56* ± *0.50*	*100*
*KC 2: To be able to stimulate and encourage individuals, their families and stakeholders regarding healthy ageing*		*3.63* ± *0.48*	*90*		*3.75* ± *0.43*	*100*		*3.75* ± *0.43*	*100*
*KC 3: To be able to advice and support individuals, families and stakeholders regarding healthy ageing / OR self-management, self-reliance and co-reliance*		*3.6* ± *0.49*	*95*		*3.69* ± *0.46*	*100*		*3.69* ± *0.46*	*100*
**HEALTH AND WELFARE ADVOCATE** **As Health and Welfare Advocates, health professionals contribute their expertise and influence when working with individuals and their families, communities or populations to promote and support healthy ageing. Health and Welfare advocacy optimizes health across the whole continuum, from the level of individuals to the population at large. The professional as Health and Welfare Advocate can influence change at any level of the continuum to enhance healthy ageing of a society**	**5**	**24**	**84**	**5**	**23**	**98**	**5**	**23**	**99.36**
*KC 1: To be able to perform a person-centered assessment of an individual focusing on the determinants of healthy ageing*	*Mean* ± *SD*	*3.42* ± *0.59*	*95*	*Mean* ± *SD*	*3.6* ± *0.49*	*100*	*Mean* ± *SD*	*3.6* ± *0.49*	*100*
*KC 2:To be able to establish a plan together with the individual, their families and relevant stakeholders to promote and support healthy ageing*		*3.44* ± *0.76*	*85*		*3.67* ± *0.47*	*100*		*3.67* ± *0.47*	*100*
*KC 3: To be able to perform actions for the promotion of healthy ageing in individuals*		*3.65* ± *0.48*	*85*		*3.67* ± *0.47*	*100*		*3.67* ± *0.47*	*100*
*KC 4: To be able to evaluate and adjust the plan on a continuing basis*		*3.59* ± *0.77*	*80*		*3.73* ± *0.44*	*100*		*3.73* ± *0.44*	*100*
*KC 5: To be able to advocate for the promotion of healthy ageing with, and on behalf of communities, populations and organizations*		*3.47* ± *0.61*	*80*		*3.53* ± *0.50*	*100*		*3.53* ± *0.50*	*100*
**LEADER** **As Leaders, health and social care professionals engage with others to contribute to a vision on healthy ageing and take responsibility for the quality of health and social care in the field of healthy ageing. They function as individual care professionals, as members of teams, and as participants and leaders in health and social care at different levels (regionally, nationally etc.)**	**3**	**11**	**77**	**3**	**11**	**99**	**3**	**11**	**98.66**
*To be able to articulate and act on both a personal vision on healthy ageing as well as a common vision shared with others*	*Mean* ± *SD*	*3.35* ± *0.59*	*80*	*Mean* ± *SD*	*3.37* ± *0.48*	*100*	*Mean* ± *SD*	*3.37* ± *0.48*	*100*
*To be able to contribute to the quality of health and social care in the domain of healthy ageing*		*3.56* ± *0.79*	*75*		*3.62* ± *0.48*	*100*		*3.62* ± *0.48*	*100*
*To be able to demonstrate leadership in the domain of healthy ageing*		*3.55* ± *0.60*	*85*		*3.44* ± *0.50*	*100*		*3.44* ± *0.50*	*100*
**PROFESSIONAL** **As Professionals, health and social care professionals are committed to the health, well-being and healthy ageing of individuals and the society through ethical practice, high personal standards of behaviour, accountability to the profession and society, physician-led regulation, and maintenance of personal health**	**2**	**8**	**91**	**2**	**8**	**97**	**2**	**8**	**98.66**
*KC 1: To be able to apply best practices and adhere to high ethical standards*	*Mean* ± *SD*	*3.79* ± *0.41*	*95*	*Mean* ± *SD*	*3.87* ± *0.33*	*100*	*Mean* ± *SD*	*3.87* ± *0.33*	*100*
*KC 2: To be able to recognize and respond to societal expectations and knowledge gaps within the healthy ageing domain*		*3.59* ± *0.49*	*85*		*3.56* ± *0.50*	*100*		*3.56* ± *0.50*	*100*
**SCHOLAR** **As scholars, health and social care professionals demonstrate a lifelong commitment to expand professional expertise in the field of healthy ageing through continuous learning. They interpret evidence based results of research and contribute to the development of knowledge and practical research in relation to the provision of care and support of individuals and their families**	**3**	**10**	**86**	**3**	**10**	**99**	**3**	**10**	**99.4**
*KC 1: To be able to engage in the continuous enhancement of their professional activities through ongoing learning*	*Mean* ± *SD*	*3.39* ± *0.59*	*85*	*Mean* ± *SD*	*3.44* ± *0.50*	*100*	*Mean* ± *SD*	*3.44* ± *0.50*	*100*
*KC 2: To be able to integrate best available evidence into practice*		*3.63* ± *0.48*	*95*		*3.81* ± *0.39*	*100*		*3.81* ± *0.39*	*100*
*KC 3: To be able to contribute to the creation and dissemination of knowledge and practices applicable to health*		*3.53* ± *0.50*	*85*		*3.44* ± *0.61*	*93*		*3.53* ± *0.50*	*100*

*KC* Key competency, *EC* Enabling competency

#### Round 1

The first round was conducted from January to February 2022. After the 22 experts agreed to participate in our study, an invitation email was sent with the link to access the online platform with the questionnaire. The first part of the questionnaire consisted of sociodemographic data. The second part included the competencies to be ranked based on the constructed competency framework. After the first round was completed, the steering group met to discuss the results and agree on the content of round two. Based on the comments of the panel of experts, some competencies were reformulated or clarified, and one new competency was included (Supplemental Online Material). For every competency, the steering group also highlighted the need to include a clear, direct question about the reasons for the choice to rate it relevant or not relevant (highlighting when the competency was rated as not relevant). Because the majority of the items achieved 75% agreement on the relevance stipulated in our criteria, in round 2, the questionnaire was sent to the experts again, including the competencies that did not reach 100% agreement, and showed the percentage of agreement achieved in round 1. The changes made to different competencies were highlighted in red.

#### Round 2

The second round was conducted from February 2022 to March 2022 and included 16 experts. The level of agreement was almost 100% in all competencies (Table 2). The recommendations, which were based on the conclusions of the steering group and agreed upon with the SIENHA Consortium for round 3, entailed reranking the competencies that did not reach 100% agreement and identifying which educational level (master’s or bachelor’s) would apply to each competency.

#### Round 3

The third round was conducted from March 2022 to April 2022, and 17 experts participated. After the completion of the three rounds, the final set of competencies consisted of 18 key competencies and 80 enabling competencies distributed across the six domains after minor adjustments agreed upon within the steering group and the consortium. These corresponded to the final competency framework proposed by the SIENHA consortium.

In terms of the recommended educational level to achieve the different competencies, the experts recommended that they should be conducted mainly at the master’s degree level in every domain.

## Discussion

To the best of our knowledge, this is the first study to describe the process of defining the competencies for health and social professionals for the promotion of healthy aging throughout the lifespan. A Delphi study was conducted to reach agreement on a competency framework, with a final consensus consisting of a set of 18 key competencies and 80 enabling competencies distributed across six domains.

The present study was conducted following a rigorous process that involved the participation of an international panel of experts with various backgrounds and extensive expertise on the topic of healthy aging, the definition of a baseline competency framework by an international, interdisciplinary team through an iterative process preceded by the elaboration of a scoping review (SIENHA, 2022), the inclusion of a steering group to monitor the entire Delphi study with mid-term discussions with the research team about consensus on different steps of the Delphi process, and adherence to international guidelines for conducting a Delphi study (Jünger et al., 2017).

Overall, the participants on the Delphi panel reached a high level of consensus on the competencies within the different domains across waves. These results are aligned with recent studies that defined the competencies in relation to medical students (Eskes et al., 2014; Robbrecht et al., 2022; Shah et al., 2020; Sohrmann et al., 2020) as well as other health practitioners, such as nurses, midwives, allied health professionals, and pharmacists (Eskes et al., 2014; Janssens et al., 2022; Sanclemente‐Dalmau et al., 2022; Westein et al., 2019). In line with some of these studies, our work had a solid foundation because it was based on the first step of a rigorous scoping review (SIENHA, 2022). Additionally, our team decided that the basis of the framework needed to be developed following a model that was previously studied and tested, the CanMEDS Framework. This is the most widely accepted and applied physician competency framework in the world and has been previously adapted to other health disciplines (Janssens et al., 2022). The definition of a competency framework does not necessarily involve following a previously defined model. However, the literature shows that the use of such a model helps to ensure the quality of the final product, with good examples found in recent work by Robbrecht (2022) and Shah et al. (2020).

The domains or dimensions embedded in our framework, which were the umbrella for the different key and enabling competencies, were the following: collaborator, communicator, health and welfare advocate, leader, professional, and scholar.

In the different dimensions, the following competencies reached 100% agreement in the first round: in the dimension of collaborator, recognizing their own roles and responsibilities related to their profession (EC 1.1); in the dimension of communicator, communicating through a person-centered approach that promotes individual trust and autonomy characterized by empathy, respect, and compassion (EC 1.1), anticipating and supporting individual and family needs (EC 1.6), and eliciting individuals’ (prior) understanding of their health issues in a non-shaming manner (EC 2.1); in the dimension of health and welfare advocate, recognizing the determinants of healthy aging (1.1); and in the dimension of professional, recognizing and responding to ethical issues and applying reflective ethical practice (EC 1.2) and demonstrating adherence to privacy and confidentiality obligations (EC 1.3). Although these competencies were a small representation of the overall framework, the fact that no disagreement existed on the part of any of the experts from the beginning of the study shows the significance of direct patient attention and care and perhaps downsizes the perspectives of health promotion and disease prevention related to healthy aging. Additionally, the analysis of the qualitative data in the different steering group discussions showed that the comments provided by the panel of experts sustained the trend of considering healthy aging from the perspective of older adults, obviating the lifespan approach. Some debate was devoted to this aspect, with the most efforts to prevent and treat the impact of aging targeting the latter stages of life (WHO, 2020). Considering that healthy aging starts from early childhood, resources should be invested to integrate its importance from the beginning of life with a primary focus on promotion and prevention (Michel et al., 2021). Our framework aims to specifically sustain this focus through the inclusion of competencies such as “working effectively with individuals and families to promote and support healthy aging throughout the lifespan”, “recognizing and understanding the impact of common risks and protective factors in relation to healthy aging”, “advocating for the promotion of healthy aging with, and on behalf of communities, populations and organizations”, and “engaging with key stakeholders to develop and sustain actions to promote healthy aging”.

In terms of agreement, no competencies reached 100% consensus in the first round in the scholar or leader dimensions. In fact, the Delphi study showed that the leader dimension achieved the least agreement, particularly during the first round (77%).

Some of the comments provided by the experts indicated that a more specific and concrete message was needed in the leader dimension, specifically in competency 1.1 (develop a specific professional vision on healthy aging), competency 1.4 (recognize opportunities to exert influence at various levels to achieve multilevel alignment in agendas and actions in the interests of individuals and their families, other professionals, organizations and society), and competency 3.1 (demonstrate abilities to lead innovative projects and demonstrate management skills for their implementation/creation). In the second round, after the adjustments suggested by the steering group and the research team, the leader dimension achieved 99% agreement.

The integration of leadership competencies has been discussed in previous work as a major challenge within the curriculum of health professionals (Binnendyk et al., 2021). In fact, some research has shown that the most frequently applied competencies in clinical practice are communicators, professionals, and collaborators (Bugaj et al., 2017). The leader dimension was introduced as an enhancement in the CanMEDS Framework (Royal College of Physicians & Surgeons of Canada, 2015). Despite the importance of leadership in health care professions, many curricular itineraries lack this specific theoretical content and are difficult to assess in the clinical setting (Maddalena, 2016; Sonnenberg et al., 2018; Steinhaeuser et al., 2013).

Due to demographic changes, it is essential that health care and social professionals have the necessary skills, attitudes and knowledge to anticipate and promote healthy aging. Students in health care and social disciplines integrate the core components of their future profession through theoretical and practical learning. In the educational field, following a competency framework for the promotion of healthy aging can help to better define and integrate the core set of skills, attitudes, knowledge and learning outcomes that integrate a vision of healthy ageing. Furthermore. teaching and assessment methodologies can be well defined and applied (Binnendyk et al., 2021; Westein et al., 2019). However, developing a competency framework is not an easy task and involves in-depth discussion and constant adaptation to the new realities within the discipline and the system (Janssens et al., 2022). Our competency framework involved an additional challenge, which was the inclusion of different professions and visions. This could be seen as a limitation, but it was balanced by the close collaboration and interprofessional work of the consortium as well as the feedback provided by the panel of experts during the consensus process. However, the analysis of the relevancy of each set of competencies for the different professions as well as the level at which each competency was located (bachelor’s or master’s) should be developed. This is part of an ongoing project within our team.

The final set of competencies can help students and educators enrich their learning and the academic content of their subjects or/and programs and can incentivize innovation. These elements can also help improve professional practice, which can directly benefit from the competency framework by developing guidelines and protocols that integrate the core axes for ethical, up-to-date and person-centered practice considering preventive and promotional focuses.

In terms of limitations, some expert profiles were missing on the panel, especially from social disciplines. The number of responses differed between rounds, with fewer participants in the second round than in the first and third rounds. However, the input of the participants was rich and well structured and helped the steering group and the consortium improve some of the competencies across the different Delphi rounds. Although the CanMEDS model is a solid foundation for the development of a new framework, it has some limitations that should be acknowledged, such as the extent to which some of the competencies described are integrated (Binnendyk et al., 2021). In the final competency framework, despite strong efforts to avoid focusing on older adults, some of the competencies might be biased, and their interpretation might lead the reader to think of the older adult population. Finally, in some Delphi studies in health care, panel members met after conducting the Delphi study. This was not the case in our study. Instead, we held multiple meetings with health and social professionals in the context of the steering group and the project consortium.

## Supplementary Information

Below is the link to the electronic supplementary material.

Supplementary file1 (DOCX 41 KB)
