# Supplementary material for: Defining a competency framework for health and social professionals to promote healthy aging throughout the lifespan: an international Delphi study
Source: Adv Health Sci Educ Theory Pract. 2024 Mar 5;29(5):1787–807. doi: 10.1007/s10459-024-10316-4 (PMC11549203; doi:10.1007/s10459-024-10316-4)
Supplement: Supplementary file 1 — Supplementary file1 (DOCX 41 KB) [file 10459_2024_10316_MOESM1_ESM.docx]

**SUPPLEMENTAL ONLINE MATERIAL**

**Table 3. Overview of results from the delphi rounds and overall consensus for each round across domains and for each competency**

| **Domains, key and enabling competencies of the SIENHA Competency Framework on healthy ageing** | **Round 1** | | **Round 2** | | **Round 3** | |
| --- | --- | --- | --- | --- | --- | --- |
| **COLLABORATOR As collaborators, health and social care professionals work together with others to promote and support healthy ageing throughout the lifespan among individuals, groups of individuals and/or communities. The forms of collaboration can involve the individuals’ families*, health and social care professionals, community partners and other stakeholders** | **Consensus**  **achieved *(relevant or very relevant)***  **(%)** | **Mean ± SD** | **Consensus**  **achieved *(relevant or very relevant)***  **(%)** | **Mean ± SD** | **Consensus**  **achieved *(relevant or very relevant)***  **(%)** | **Mean ±**  **SD** |
| Key competence 1: To be able to work effectively with other professionals within and outside the health and social care profession to promote and support healthy ageing throughout the lifespan | 90.47 | 3.79 **±** 0.41 | 100 | 3.87 ± 0.33 | 100 | 3.87 ± 0.33 |
| *Enabling competence 1.1: recognize their own roles and responsibilities related to their profession* | 100 | 3.71 **±** 0.45 | 100 | 3.71 ± 0.45 | 100 | 3.71 ± 0.45 |
| *Enabling competence 1.2: recognize the roles and responsibilities of other professionals and organizations working within the domain of healthy ageing* | 95.23 | 3.5 **±** 0.5 | 100 | 3.75 ± 0.43 | 100 | 3.75 ± 0.43 |
| *Enabling competence 1.3: negotiate overlapping and shared responsibilities with other professionals* | 90.47 | 3.58 **±** 0.49 | 93 | 3.5 ± 0.61 | 100 | 3.47 ± 0.50 |
| *Enabling competence 1.4: work collaboratively with other professionals and organizations to support and promote healthy ageing* | 95.23 | 3.6 **±** 0.49 | 100 | 3.56 ± 0.50 | 100 | 3.56 ± 0.50 |
| *Enabling competence 1.5: establish and maintain open and honest professional relationships, making referrals to other professionals, as necessary* | 95.23 | 3.6 **±** 0.49 | 100 | 3.62 ± 0.48 | 100 | 3.62 ± 0.48 |
| *Enabling competence 1.6: Use innovative context and person-specific methods of collaboration.*  *NOTE: Innovation can be considered as the application of ideas that are novel and useful for a given context and person (coming up to new ways of doing things that can help overcome difficulties that appear in the current way of doing things)*  ***Round 2:*** *Use context and person-specific methods of collaboration, considering innovative approaches.*  *NOTE: Innovation can be considered as the application of ideas that are novel and useful for a given context and person (coming up to new ways of doing things that can help overcome difficulties that appear in the current way of doing things)*  ***Round 1:*** *use innovative methods of collaboration* | *76.19* | 3.29 **±** 0.57 | 100 | 3.31 **±** 0.46 | 100 | 3.35 **±** 0.48 |
| *Enabling competence 1.7: use communication technologies and health technology when collaborating with other professionals* | 71.42 | 3.4 **±** 0.49 | 100 | 3.31 ± 0.46 | 100 | 3.31 ± 0.46 |
| Key competence 2: To be able to work effectively with individuals and families* to promote and support healthy ageing throughout the lifespan | 80.95 | 3.67 **±** 0.74 | 100 | 3.75 ± 0.43 | 100 | 3.75 ± 0.43 |
| *Enabling competence 2.1: work with individuals’ families on a basis of respect and equity* | 95.23 | 3.8 ± 0.40 | 100 | 3.81 ± 0.39 | 100 | 3.81 ± 0.39 |
| *Enabling competence 2.2: engage in respectful shared decision-making with individuals’ and their families* | 95.23 | 3.67 ± 0.71 | 100 | 3.81 ± 0.39 | 100 | 3.81 ± 0.39 |
| *Enabling competence 2.3: coach families to enable them to support the individual in healthy ageing* | 85.71 | 3.47 ± 0.75 | 93 | 3.56 ± 0.61 | 100 | 3.53 ± 0.50 |
| *Enabling competence 2.4: facilitate families’ adaptive capacity related to healthy ageing* | 85.71 | 3.42 ± 0.75 | 93 | 3.56 ± 0.61 | 100 | 3.41 ± 0.60 |

** The term “families” relates and includes all of those personally significant to the individual who are involved with his or her life, including family members, partners, informal caregivers, legal guardians, and substitute decision-makers*

|  | **R1** | | **R2** | | **R3** | |
| --- | --- | --- | --- | --- | --- | --- |
| **COMMUNICATOR As Communicators, health and social care professionals form positive relationships with the individuals and their families, facilitating the gathering and sharing of essential information related to healthy ageing. Using person-centered communication, they support and advise individuals in shared decision-making and lead effective interactions that promote health and well-being** | **Consensus**  **achieved *(relevant or very relevant)***  **(%)** | **Mean ± SD** | **Consensus**  **achieved *(relevant or very relevant)***  **(%)** | **Mean ± SD** | **Consensus**  **achieved *(relevant or very relevant)***  **(%)** | **Mean ±**  **SD** |
| Key competence 1: To be able to communicate effectively with individuals, families and stakeholders to establish strong positive relationships with them.  **Round 1:** THE PROFESSIONALS ARE ABLE TO establish strong positive relationships with individuals and their families, coupled with effective communication | 90 | 3.63 ± 0.48 | 100 | 3.56 ± 0.50 | 100 | 3.56 ± 0.50 |
| *Enabling competence 1.1: communicate through a person-centered approach that promotes individual trust and autonomy, characterized by empathy, respect, and compassion* | 100 | 3.71 ± 0.45 | 100 | 3.71 ± 0.45 | 100 | 3.71 ± 0.45 |
| *Enabling competence 1.2: respect all kinds of diversity in the relationship with individuals, families and stakeholders*  **Round 1:** *respect individual and cultural diversity in delivered care and services* | 95 | 3.55 ± 0.50 | 100 | 3.56 ± 0.50 | 100 | 3.56 ± 0.50 |
| *Enabling competence 1.3: create a shame-free environment and respond to emotions within the frame of an ethical relationship*  ***Round 1****: create a shame-free environment and respond to emotions* | 86 | 3.61 ± 0.49 | 93 | 3.75 ± 0.56 | 100 | 3.41 ± 0.49 |
| *Enabling competence 1.4: communicate clearly through plain language, avoidance of jargon and prioritization of information* | 95 | 3.8 ± 0.4 | 100 | 3.75 ± 0.43 | 100 | 3.75 ± 0.43 |
| *Enabling competence 1.5: share information and explanations that are clear, accurate and timely* | 90 | 3.6 ± 0.73 | 100 | 3.75 ± 0.43 | 100 | 3.75 ± 0.43 |
| *Enabling competence 1.6: anticipate and support individual and family needs* | 100 | 3.62 ± 0.49 | 100 | 3.62 ± 0.49 | 100 | 3.62 ± 0.49 |
| Key competence 2: To be able to stimulate and encourage individuals, their families and stakeholders regarding healthy ageing | 90 | 3.63 ± 0.48 | 100 | 3.75 ± 0.43 | 100 | 3.75 ± 0.43 |
| *Enabling competence 2.1: elicit individuals (prior) understanding of their health issues in a non-shaming manner* | 100 | 3.52 ± 0.50 | 100 | 3.52 ± 0.50 | 100 | 3.52 ± 0.50 |
| *Enabling competence 2.2: share and discuss relevant information based on analysis of assessments results* | 90 | 3.42 ± 0.49 | 100 | 3.37 ± 0.48 | 100 | 3.37 ± 0.48 |
| *Enabling competence 2.3: use explanations that are clear, accurate, and timely* | 95 | 3.75 ± 0.43 | 100 | 3.81 ± 0.39 | 100 | 3.81 ± 0.39 |
| *Enabling competence 2.4: respond to wishes, preferences and discuss possibilities to self-manage* | 95 | 3.75 ± 0.43 | 100 | 3.69 ± 0.46 | 100 | 3.69 ± 0.46 |
| *Enabling competence 2.5: encourage individuals and their family to ask questions* | 86 | 3.53 ± 0.75 | 93 | 3.56 ± 0.61 | 100 | 3.71 ± 0.46 |
| Key competence 3: To be able to advice and support individuals, families and stakeholders regarding healthy ageing / OR self-management, self-reliance and co-reliance | 95 | 3.6 ± 0.49 | 100 | 3.69 ± 0.46 | 100 | 3.69 ± 0.46 |
| *Enabling competence 3.1: strengthen self-management and resilience of individual* | 90 | 3.6 ± 0.73 | 100 | 3.81 ± 0.39 | 100 | 3.81 ± 0.39 |
| *Enabling competence 3.2: to advise and accompany the older adult in processes of socio-educational development*  ***Round 1****: to advise and accompany the elderly in processes of socio-educational development* | 81 | 3.28 ± 0.56 | 93 | 3.25 ± 0.56 | 88 | 3.12 ± 0.58 |
| *Enabling competence 3.3: to offer advice on individual and collective learning needs*  ***Round 1:*** *to offer advice on individual learning needs* | 76 | 3.41 **±** 0.77 | 93 | 3.44 **±** 0.61 | 94 | 3.35 **±** 0.59 |
| *Enabling competence 3.4: help individuals to identify and address facilitators and possible barriers* | 86 | 3.35 **±** 0.79 | 93 | 3.56 **±** 0.61 | 100 | 3.59 **±** 0.49 |
| *Enabling competence 3.5: consider different coping strategies for individuals struggling with changes* | 90 | 3.48 **±** 0.79 | 100 | 3.69 ± 0.46 | 100 | 3.69 ± 0.46 |
| *Enabling competence 3.6: create partnerships with individuals, families and stakeholders for long-term support*  ***Round 1:*** *create partnerships for long-term support* | 80 | 3.59 **±** 0.77 | 93 | 3.69 **±** 0.58 | 100 | 3.65 **±** 0.48 |

|  | **R1** | | **R2** | | **R3** | |
| --- | --- | --- | --- | --- | --- | --- |
| **HEALTH AND WELFARE ADVOCATE As Health and Welfare Advocates, health professionals contribute their expertise and influence when working with individuals and their families, communities or populations to promote and support healthy ageing. Health and Welfare advocacy optimizes health across the whole continuum, from the level of individuals to the population at large. The professional as Health and Welfare Advocate can influence change at any level of the continuum to enhance healthy ageing of a society** | **Consensus**  **achieved *(relevant or very relevant)***  **(%)** | **Mean ± SD** | **Consensus**  **achieved *(relevant or very relevant)***  **(%)** | **Mean ± SD** | **Consensus**  **achieved *(relevant or very relevant)***  **(%)** | **Mean ±**  **SD** |
| Key competence 1: To be able to perform a person-centered assessment of an individual focusing on the determinants of healthy ageing | 95 | 3.42 ± 0.59 | 100 | 3.6 ± 0.49 | 100 | 3.6 ± 0.49 |
| *Enabling competence 1.1: recognize determinants of healthy ageing* | 100 | 3.65 ± 0.48 | 100 | 3.65 ± 0.48 | 100 | 3.65 ± 0.48 |
| *Enabling competence 1.2: recognize and understand the impact of common risks and protective factors in relation to healthy ageing* | 95 | 3.55 ± 0.74 | 100 | 3.8 ± 0.4 | 100 | 3.8 ± 0.4 |
| *Enabling competence 1.3: perform a screening for physical, mental, environmental and social factors that influence healthy ageing*  ***Round 1****: perform a screening for physical, mental and social factors that influence healthy ageing* | 85 | 3.53 ± 0.94 | 100 | 3.8 ± 0.4 | 100 | 3.8 ± 0.4 |
| *Enabling competence 1.4: apply knowledge of the clinical, biomedical and social sciences relevant to healthy ageing* | 70 | 3.56 ± 0.86 | 100 | 3.87 ± 0.34 | 100 | 3.87 ± 0.34 |
| *Enabling competence 1.5: assess individual norms and values informed by culture, religion, spirituality, intergenerational differences, and sexual diversity* | 75 | 3.56 ± 0.79 | 100 | 3.67 ± 0.47 | 100 | 3.67 ± 0.47 |
| *Enabling competence 1.6: recognize modifiable or treatable factors concerning healthy ageing* | 85 | 3.42 ± 0.94 | 100 | 3.67 ± 0.47 | 100 | 3.67 ± 0.47 |
| *Enabling competence 1.7: identify main problems together with the individual, their families and relevant stakeholders* | 85 | 3.61 ± 0.49 | 100 | 3.87 ± 0.34 | 100 | 3.87 ± 0.34 |
| *Enabling competence 1.8: assess the individual, their families and relevant stakeholders sense of agency and skills for behavior change* ***(new competency appeared in round 2)*** | - | - | 93 | 3.33 **±** 0.60 | 88 | 3.29 **±** 0.66 |
| Key competence 2: To be able to establish a plan together with the individual, their families and relevant stakeholders to promote and support healthy ageing | 85 | 3.44 ± 0.76 | 100 | 3.67 ± 0.47 | 100 | 3.67 ± 0.47 |
| *Enabling competence 2.1: formulate goals based on analysis of the assessment results* | 80 | 3.53 ± 0.78 | 100 | 3.8 ± 0.4 | 100 | 3.8 ± 0.4 |
| *Enabling competence 2.2: engage in respectful shared decision-making, considering needs and preferences of the individual* | 90 | 3.72 ± 0.45 | 100 | 3.87 ± 0.34 | 100 | 3.87 ± 0.34 |
| *Enabling competence 2.3: develop a plan based on the health and social status of the individual, as well as their values, preferences and resource for behavior change*  ***Round 2:*** *develop a plan based on the health and social status of the individual, as well as their resources for behavior change*  ***Round 1:*** *develop a plan based on the health and social status of the individual* | 80 | 3.59 ± 0.77 | 87 | 3.47 ± 0.88 | 94 | 3.47 ± 0.61 |
| *Enabling competence 2.4: develop a plan that includes both actions within and outside one’s own profession* | 90 | 3.42 ± 0.59 | 92 | 3.47 ± 0.62 | 100 | 3.65 ± 0.48 |
| Key competence 3: To be able to perform actions for the promotion of healthy ageing in individuals | 85 | 3.65 ± 0.48 | 100 | 3.67 ± 0.47 | 100 | 3.67 ± 0.47 |
| *Enabling competence 3.1: facilitate the individuals, families and relevant stakeholders' active participation* | 90 | 3.55 ± 0.50 | 100 | 3.6 ± 0.49 | 100 | 3.6 ± 0.49 |
| *Enabling competence 3.2: carry out a tailor made program to promote and support healthy ageing* | 90 | 3.67 ± 0.47 | 100 | 3.73 ± 0.44 | 100 | 3.73 ± 0.44 |
| *Enabling competence 3.3: carry out professional actions demonstrating knowledge and skills in the field of healthy ageing* | 75 | 3.73 ± 0.44 | 93 | 3.67 ± 0.60 | 100 | 3.53 ± 0.50 |
| *Enabling competence 3.4: be able to apply technologies to support the professional actions*  ***Round 1:*** *apply technologies to support the professional actions* | 70 | 3.33 ± 0.79 | 100 | 3.53 ± 0.50 | 100 | 3.53 ± 0.50 |
| Key competence 4: To be able to evaluate and adjust the plan on a continuing basis | 80 | 3.59 ± 0.77 | 100 | 3.73 ± 0.44 | 100 | 3.73 ± 0.44 |
| *Enabling competence 4.1: monitor the situation of the individual* | 80 | 3.5 ± 0.83 | 93 | 3.73 ± 0.57 | 100 | 3.71 ± 0.46 |
| *Enabling competence 4.2: monitor the progress of the goals set and adjust and change the plan when needed* | 80 | 3.53 ± 0.78 | 100 | 3.67 ± 0.47 | 100 | 3.67 ± 0.47 |
| Key competence 5: To be able to advocate for the promotion of healthy ageing with, and on behalf of communities, populations and organizations | 80 | 3.47 ± 0.61 | 100 | 3.53 ± 0.50 | 100 | 3.53 ± 0.50 |
| *Enabling competence 5.1: raise awareness of and influence public opinion regarding healthy ageing* | 85 | 3.55 ± 0.60 | 100 | 3.67 ± 0.47 | 100 | 3.67 ± 0.47 |
| *Enabling competence 5.2: work with communities, populations and organizations to identify the determinants and health/social inequalities that affect healthy ageing*  ***Round 1:*** *work with communities, populations and organizations to identify the determinants that affect healthy ageing* | 90 | 3.63 ± 0.58 | 100 | 3.67 ± 0.47 | 100 | 3.67 ± 0.47 |
| *Enabling competence 5.3: engage with key stakeholders to develop and sustain actions to promote healthy ageing* | 85 | 3.65 ± 0.48 | 100 | 3.6 ± 0.49 | 100 | 3.6 ± 0.49 |
| *Enabling competence 5.4: design and evaluate clear and locally tailored actions to address stakeholder problems and needs* | 80 | 3.56 ± 0.50 | 100 | 3.53 ± 0.50 | 100 | 3.53 ± 0.50 |
| *Enabling competence 5.5: contribute to prevention and health literacy of communities* | 80 | 3.53 ± 0.61 | 100 | 3.67 ± 0.47 | 100 | 3.67 ± 0.47 |
| *Enabling competence 5.6: contribute to the process of promoting healthy ageing in the community or population they serve* | 80 | 3.28 ± 0.8 | 100 | 3.47 ± 0.50 | 100 | 3.47 ± 0.50 |
| *Enabling competence 5.7: communicate campaigns to promote healthy ageing working with communication professionals (journalists, marketing, etc.)*  ***Round 1:*** *communicate campaigns to promote healthy ageing* | 80 | 3.22 ± 0.79 | 100 | 3.47 ± 0.50 | 100 | 3.47 ± 0.50 |

|  | **R1** | | **R2** | | **R3** | |
| --- | --- | --- | --- | --- | --- | --- |
| **LEADER As Leaders, health and social care professionals engage with others to contribute to a vision on healthy ageing and take responsibility for the quality of health and social care in the field of healthy ageing. They function as individual care professionals, as members of teams, and as participants and leaders in health and social care at different levels (regionally, nationally etc.)** | **Consensus**  **achieved *(relevant or very relevant)***  **(%)** | **Mean ± SD** | **Consensus**  **achieved *(relevant or very relevant)***  **(%)** | **Mean ± SD** | **Consensus**  **achieved *(relevant or very relevant)***  **(%)** | **Mean ±**  **SD** |
| Key competence 1: To be able to articulate and act on both a personal vision on healthy ageing as well as a common vision shared with others | 80 | 3.35 ± 0.59 | 100 | 3.37 ± 0.48 | 100 | 3.37 ± 0.48 |
| *Enabling competence 1.1: develop a specific professional vision on healthy ageing*  ***Round 2:*** *develop a specific professional vision on healthy ageing according to their work, speciality*  ***Round 1:*** *develop a personal vision on healthy ageing* | 70 | 3.47 ± 0.62 | 93 | 3.5 ± 0.61 | 94 | 3.37 ± 0.60 |
| *Enabling competence 1.2: develop a shared vision together with others within the domain of healthy ageing* | 75 | 3.47 ± 0.50 | 93 | 3.37 ± 0.60 | 94 | 3.62 ± 0.60 |
| *Enabling competence 1.3: promote their vision and build support so that others participate from a common interest* | 75 | 3.6 ± 0.49 | 93 | 3.44 ± 0.61 | 94 | 3.37 ± 0.60 |
| *Enabling competence 1.4: recognize the opportunities to exert influence at various levels achieving multilevel alignment in agendas and actions, in the interests of individuals and their families, other professionals, organizations and society*  ***Round 1:*** *recognize the opportunities to exert influence at various levels, in the interests of individuals and their families, other professionals, organizations and society* | 70 | 3.53 ± 0.80 | 100 | 3.56 ± 0.50 | 100 | 3.56 ± 0.50 |
| Key competence 2: To be able to contribute to the quality of health and social care in the domain of healthy ageing | 75 | 3.56 ± 0.79 | 100 | 3.62 ± 0.48 | 100 | 3.62 ± 0.48 |
| *Enabling competence 2.1: lead and participate in activities that promote quality of care in the domain of healthy ageing* | 75 | 3.44 ± 0.61 | 100 | 3.56 ± 0.50 | 100 | 3.56 ± 0.50 |
| *Enabling competence 2.2: improve daily practice by applying a process of continuous quality improvement* | 80 | 3.53 ± 0.61 | 100 | 3.5 ± 0.50 | 100 | 3.5 ± 0.50 |
| *Enabling competence 2.3: contribute to the development of systems, policies and procedures for the promotion of healthy ageing* | 80 | 3.56 ± 0.50 | 100 | 3.5 ± 0.5 | 100 | 3.5 ± 0.5 |
| Key competence 3: To be able to demonstrate leadership in the domain of healthy ageing | 85 | 3.55 ± 0.60 | 100 | 3.44 ± 0.50 | 100 | 3.44 ± 0.50 |
| *Enabling competence 3.1: demonstrate abilities to lead innovation projects and demonstrate managment skills to apply their implementation/creation*  ***Round 1:*** *demonstrate leadership skills to enhance innovation projects* | 70 | 3.57 ± 0.49 | 100 | 3.44 ± 0.50 | 100 | 3.44 ± 0.50 |
| *Enabling competence 3.2: facilitate change in the field of healthy ageing to enhance services and outcomes* | 85 | 3.41 ± 0.49 | 100 | 3.31 ± 0.46 | 100 | 3.31 ± 0.46 |
| *Enabling competence 3.3: share knowledge and contribute to learning opportunities within the healthy ageing domain* | 85 | 3.28 **±** 0.73 | 100 | 3.31 ± 0.46 | 100 | 3.31 ± 0.46 |
| *Enabling competence 3.4: exert influence despite barriers such as hierarchical relationships, divergent interests, beliefs of others or own emotions* | 75 | 3.31 **±** 0.77 | 100 | 3.37 ± 0.48 | 100 | 3.37 ± 0.48 |

|  | **R1** | | **R2** | | **R3** | |
| --- | --- | --- | --- | --- | --- | --- |
| **PROFESSIONAL As Professionals, health and social care professionals are committed to the health, well-being and healthy ageing of individuals and the society through ethical practice, high personal standards of behaviour, accountability to the profession and society, physician-led regulation, and maintenance of personal health.** | **Consensus**  **achieved *(relevant or very relevant)***  **(%)** | **Mean ± SD** | **Consensus**  **achieved *(relevant or very relevant)***  **(%)** | **Mean ± SD** | **Consensus**  **achieved *(relevant or very relevant)***  **(%)** | **Mean ±**  **SD** |
| Key competence 1: To be able to apply best practices and adhere to high ethical standards | 95 | 3.79 ± 0.41 | 100 | 3.87 ± 0.33 | 100 | 3.87 ± 0.33 |
| *Enabling competence 1.1: apply evidence-based practice in the field of healthy ageing* | 95 | 3.65 ± 0.73 | 100 | 3.87 ± 0.33 | 100 | 3.87 ± 0.33 |
| *Enabling competence 1.2: recognize and respond to ethical issues and apply a reflective ethical practice* | 100 | 3.75 ± 0.43 | 100 | 3.75 ± 0.43 | 100 | 3.75 ± 0.43 |
| *Enabling competence 1.3: demonstrate adhere to privacy and confidentiality obligations* | 100 | 3.6 ± 0.49 | 100 | 3.6 ± 0.49 | 100 | 3.6 ± 0.49 |
| *Enabling competence 1.4: demonstrate equality in the treatment and combat ageism* | 95 | 3.68 ± 0.46 | 100 | 3.75 ± 0.43 | 100 | 3.75 ± 0.43 |
| Key competence 2: To be able to recognize and respond to societal expectations and knowledge gaps within the healthy ageing domain | 85 | 3.59 ± 0.49 | 100 | 3.56 ± 0.50 | 100 | 3.56 ± 0.50 |
| *Enabling competence 2.1: recognize implications of health inequalities and inequities for active and healthy ageing with reference to social determinants of health and act to prevent them* | 85 | 3.44 ± 0.60 | 100 | 3.5 ± 0.5 | 100 | 3.5 ± 0.5 |
| *Enabling competence 2.2: reveal uncertainties and knowledge gaps in practice, which relate to environments and processes that support healthy aging in the long term* | 90 | 3.44 ± 0.50 | 100 | 3.5 ± 0.50 | 100 | 3.5 ± 0.50 |
| *Enabling competence 2.3: consider jurisdictions and legal frameworks for healthy ageing (from both a national and an international perspective)*  ***Round 1:*** *consider jurisdictions and legal frameworks for healthy ageing (from an international perspective)* | 80 | 3.28 **±** 0.73 | 75 | 3.25 **±** 0.75 | 87 | 3.33 **±** 0.70 |
| *Enabling competence 2.4: develop and promote actions to the senior populations within the scope of health, education and psychosocial fields* | 85 | 3.44 ± 0.60 | 100 | 3.69 ± 0.46 | 100 | 3.69 ± 0.46 |

|  | **R1** | | **R2** | | **R3** | |
| --- | --- | --- | --- | --- | --- | --- |
| **SCHOLAR As scholars, health and social care professionals demonstrate a lifelong commitment to expand professional expertise in the field of healthy ageing through continuous learning. They interpret evidence based results of research and contribute to the development of knowledge and practical research in relation to the provision of care and support of individuals and their families.** | **Consensus**  **achieved *(relevant or very relevant)***  **(%)** | **Mean ± SD** | **Consensus**  **achieved *(relevant or very relevant)***  **(%)** | **Mean ± SD** | **Consensus**  **achieved *(relevant or very relevant)***  **(%)** | **Mean ±**  **SD** |
| Key competence 1: To be able to engage in the continuous enhancement of their professional activities through ongoing learning | 85 | 3.39 ± 0.59 | 100 | 3.44 ± 0.50 | 100 | 3.44 ± 0.50 |
| *Enabling competence 1.1: develop, implement, monitor, and revise a personal learning plan to enhance concepts and theories of ageing* | 80 | 3.62 ± 0.48 | 100 | 3.56 ± 0.50 | 100 | 3.56 ± 0.50 |
| *Enabling competence 1.2: generate educational contexts that promote learning and advocate lifelong education to meet the needs to ageing* | 85 | 3.47 ± 0.50 | 100 | 3.44 ± 0.50 | 100 | 3.44 ± 0.50 |
| *Enabling competence 1.3: demonstrate educational and research skills* | 75 | 3.29 ± 0.66 | 81 | 3.19 ± 0.73 | 100 | 3.4 ± 0.49 |
| Key competence 2: To be able to integrate best available evidence into practice | 95 | 3.63 ± 0.48 | 100 | 3.81 ± 0.39 | 100 | 3.81 ± 0.39 |
| *Enabling competence 2.1: apply evidence-based practice for health and wellbeing of the older people* | 95 | 3.58 ± 0.49 | 100 | 3.75 ± 0.43 | 100 | 3.75 ± 0.43 |
| *Enabling competence 2.2: support the transfer of evidence based practice needs to be present for the general population and for all actors of the health sector* | 85 | 3.55 ± 0.76 | 100 | 3.81 ± 0.39 | 100 | 3.81 ± 0.39 |
| *Enabling competence 2.3: demonstrate basic health data analysis skills* | 80 | 3.5 ± 0.5 | 100 | 3.44 ± 0.50 | 100 | 3.44 ± 0.50 |
| Key competence 3: To be able to contribute to the creation and dissemination of knowledge and practices applicable to health | 85 | 3.53 ± 0.50 | 93 | 3.44 ± 0.61 | 100 | 3.53 ± 0.50 |
| *Enabling competence 3.1: develop and promote actions for the senior populations within the scope of health, education and psychosocial fields* | 85 | 3.41 ± 0.49 | 100 | 3.37 ± 0.48 | 100 | 3.37 ± 0.48 |
| *Enabling competence 3.2: identify the medium to present the education in order to reach a wide audience* | 70 | 3.37 ± 0.70 | 81 | 3.31 ± 0.77 | 93 | 3.2 ± 0.54 |
| *Enabling competence 3.3: integrate and transfer research into practice* | 85 | 3.59 ± 0.49 | 100 | 3.75 ± 0.43 | 100 | 3.75 ± 0.43 |
| *Enabling competence 3.4: be forward looking to create change in terms of existing patterns, behaviour, and mindsets* | 85 | 3.59 ± 0.49 | 100 | 3.69 ± 0.46 | 100 | 3.69 ± 0.46 |
